# CPRMethicillin resistant coagulase-negative staphylococci isolated from South Korean ducks exhibiting tremor

**DOI:** 10.1186/1751-0147-55-88

**Published:** 2013-12-11

**Authors:** Jee Eun Han, Sun Young Hwang, Ji Hyung Kim, Sang Phil Shin, Jin Woo Jun, Ji Young Chai, Yong Ho Park, Se Chang Park

**Affiliations:** 1 College of Veterinary Medicine and Research Institute for Veterinary Science, Seoul National University, Seoul 151-742, Republic of Korea; 2 Korea Institute of Ocean Science & Technology, Ansan 426-744, Republic of Korea; 3 Department of Rheumatology, Bundang Jesaeng Hospital, Seongnam 463-774, Republic of Korea

**Keywords:** Methicillin resistance, Coagulase negative staphylococci, Duck, SCC*mec* complex

## Abstract

**Background:**

We describe coagulase-negative staphylococci (CoNS) isolates collected from ducklings exhibiting tremor in South Korea over the period of 2010 to 2011. Screening of antimicrobial susceptibility and analysis of SCC*mec* elements of CoNS were also investigated.

**Results:**

*Staphylococcus cohnii* was the most frequent staphylococcus (9 isolates) and *S. sciuri* (4 isolates), *S. lentus* (3 isolate), *S. simulans* (1 isolate) and *S. epidermidis* (1 isolate) were also detected. Among the 15 antimicrobials tested in this study, resistance against oxacillin (15 isolates, 83.3%) was most frequently observed, but only one isolate (SNUDS-1) possessed *mecA.* This isolate was shown to possess SCC*mec* type III; the type 3 *ccr* complex and the class A *mec* complex.

**Conclusions:**

Based on these results, isolate SNUDS-1 was shown to possess SCC*mec* type III; the type 3 *ccr* complex and the class A *mec* complex. Although the SCC*mec* type III is not predominant in human, MR-CoNS (Methicillin resistance Coagulase-negative staphylococci) in food animals should be monitored to prevent the dissemination of antimicrobial resistance genes and resistant pathogens to the community.

## Background

Coagulase-negative staphylococci (CoNS) are commensal bacterial species and opportunistic pathogens that can cause infections in human and animals [1]. CoNS are considered a reservoir of antimicrobial resistance since they usually possess various antimicrobial resistance-associated determinants [2]. Methicillin resistance (MR) is one of the most serious public health issues and can increase both the failure rate of antibiotic therapy and mortality rates in human diseases [3]. MR-CoNS were also found in animals with clinical infections. Van Duijkeren *et al*. detected MR *Staphylococcus haemolyticus* from cystitis, rhinitis, bronchitis and pyoderma in cats and dogs [4]. Fessler *et al*. [5] isolated different MR-CoNS species from bovine mastitis. However, MR-CoNS were rarely reported in avian disease, but rather usually reported from healthy birds [6].

The determinant of MR is *mecA*, which encodes a penicillin-biinding protein (PBP2a) that has a low affinity to beta-lactam antibiotics. This gene is located on the staphyloccoccal cassette chromosome *mec* (SCC*mec*) element, a genomic island ubiquitously disseminated among staphylococci [7]. The SCC*mec* element carries two essential parts, the *mec* gene complex consist of *mecA* and its regulator genes and the cassette chromosome recombinase (*ccr*) complex composed of recombinase genes. The regions other than those two complexes are designated junkyard (J) regions. The SCC*mec* element is typed by its *mecA* and *ccr* complex and the J regions, which define the subtype of the element [8]. Although the origin of the SCC*mec* elements remains unknown, it has been suggested that the SCC*mec* elements evolved in CoNS and then were horizontally transferred among different *Staphylococcus* species [9]. In particular, *S. fleurettii, S. sciuri* and *S. vitulinus* are suggested as a origin of the *mecA* element of SCC*mec* elements [10].

There is concern that CoNS in food animals can be disseminated to humans via the food production chain [11]. Furthermore, the SCC*mec* elements in MR-CoNS of food animals are transmissible to humans via food [12]. Therefore, screening the antimicrobial resistance and analysis of SCC*mec* elements of CoNS in food animals is important for public health. Despite the importance of such a screen, many researchers have only focused on MR *S. aureus* (MRSA), the most pathogenic staphylococci, and few data are available for MR-CoNS among the ducks. In this study, we isolated CoNS isolates from ducklings and investigated their antimicrobial susceptibility. In addition, the SCC*mec* element of the isolated MR-CoNS isolate was analyzed.

## Methods

### Bacterial isolation

Samples were collected from ducklings exhibiting clinical signs of listlessness, ataxia, tremors of the head and legs, and coma, originated from 4 different farms in South Korea over the period of 2010 to 2011. Swabs from the organs were streaked on 5% sheep blood agar and incubated for 24–48 hr at 37°C. After incubation, among the representative colony of the plate, one staphylococci-like colony was collected as described by Moon *et al*. [13]. The isolates were stored in tryptic soy broth (TSB; Difco, USA) with 10% glycerol at -80°C until used. College of Veterinary Medicine is certificated institution for experiments using laboratory animals and we carried out the experiment using ethical way. In this experiment, moribund samples were anesthetized according to the established rules approved by the Seoul National University Institutional Animal Care and Use Committee.

### DNA extraction

Bacterial stock was sub-cultured on tryptic soy agar (TSA; Difco) at for 24–48 hr at 37°C and then single colony was sub-cultured in TSB for DNA extraction. Chromosomal DNA isolation was performed using the Wizard genomic DNA purification kit (Promega, Madison, USA) according to the manufacture’s instruction except using lysostaphin (0.5 mg/ml) and RNase (0.3 mg/ml) for the lysis step.

### Bacterial identification

Bacterial species were confirmed by species-specific polymerase chain reaction (PCR) and sequencing analysis of the *sodA* gene for CNS as described by Poyart *et al*. [14]. Sequencing was carried out by the Macrogen Genomic Division, Korea. Analyzed sequence was aligned with full genome sequenced *Staphylococcus* species using the NCBI BLAST (http://blast.ncbi.nlm.nih.gov/) to confirm their species when the similarity was over 99%.

### Antimicrobial susceptibility test

Antimicrobial susceptibility tests for staphylococcal isolates were carried out using the disk diffusion method according to the Clinical Laboratory Standard Institute guidelines [15,16]. Fifteen antimicrobials were used: cefoxitin, ciprofloxacin, clindamycin, erythromycin, gentamicin, linezolid, mupirocin, oxacillin, penicillin, quinupristin/dalfopristin, rifampicin, teicoplanin, tetracycline, trimethoprim/sulfamethoxazole and vancomycin. The minimum inhibitory concentration (MIC) of oxacillin was determined using the broth microdilution method [15,16]. Isolates that had a MIC ≥ 0.5 μg/ml were defined as oxacillin resistant isolates. For quality control, *S. aureus* ATCC25923 and *S. aureus* ATCC29213 were used for the disc diffusion tests and the microdilution test respectively.

### Multiplex PCR

For detection of *mecA*, PCR followed by sequencing was performed as described by Zhang *et al*. [17]. For SCC*mec* element typing, the *mec* gene and *ccr* complexes were identified by multiplex PCR and subsequent sequencing as described by Kondo *et al*. [18], who detected the class A, B, C1 and C2 *mec* complex. Also, *ccrA*, *ccrB* and *ccrC* were detected as descrbed by Zong *et al*. [19] and Zong and Lu [20] and Pi *et al*. [21] (Table 1).

**Table 1:** Primers used in this study

**Primer**	Nucleotide sequence^* ^**(5′-3′)**	**Target location**	**Reference**
***sodA***			
**d1**	CCITAYICITAYGAYGCIYTIGARCC	*sodA*	[14]
**d2**	ARRTARTAIGCRTGYTCCCAIACRTC		
***mec *** **complex**			
**mA7**	ATATACCAAACCCGACAACTACA	*mecA*	[18]
**mI6**	CATAACTTCCCATTCTGCAGATG	*mecI*	
**IS7**	ATGCTTAATGATAGCATCCGAATG	IS*1272*	
**IS2 (iS-2)**	TGAGGTTATTCAGATATTTCGATGT	IS*431*	
***ccr *** **complex**			
**ccrA-UF1**	AATGTGAHGTATTATGTTGYTA	*ccrA*	[20]
**ccrA-UR1**	GGTTCATTTTTDAARTAGAT		
**ccrA-UF2**	AYTHCATCGYAAYYTGAAAAA	*ccrA*	[19]
**ccrA-UR2**	ACGDCCACARTAGTTAGGRTT		
**ccrA_up**	TGCATTCATGTTTTGAGGAC	*ccrA*	[21]
**ccrA_dw**	CAATGTGACGTATTGTGTTG		
**ccrB-UF1**	CGTGTATCAACDGAAATVCAA	*ccrB*	[20]
**ccrB-UR1**	CTTTATCACTTTTGAYWATTTC		
**ccrB_up**	GTTCCTTTACCATGGACTTG	*ccrB*	[21]
**ccrB_dw**	CTAGAAGGCTACTATCAAGG		
**ccrC-UF1**	GCAATGAAACGTCTATTACAA	*ccrC1*	[19]
**ccrC-UR1**	TTTCATCRATAACYAAATCA		
**28-24**	GGAACAATCAGAGCGTGGA	*ccrC2*	[20]
**28-26**	ACGTTTCACAGCCCAATTTT		

^*^D: A, G or T; H: H, C or T; M: A or C; R: A or G; W: A or T; Y: C or T; V: A, C or G.

## Results

### Isolation and identification of CoNS

Organ samples (trachea, lung and brain tissue) were collected from 55 ducklings displaying clinical signs of tremors. A total of 18 isolates were collected from different animals and identified as CoNS (Table 2). The staphylococcal isolation rate was 32.7% and the most frequently isolated species was *S. cohnii.*

**Table 2:** Coagulase-negative staphylococcal isolates isolated from duck with tremor

**Isolate**	**Organ**	**Species**	***mecA***	Antimicrobial resistant pattern^*^	**Oxacillin**	
					**MIC (μg/ml)**	**Interpretation**
**SNUDS-2**	Brain	*S. cohnii*	-	Cef-Pen-Oxa-Cip-SxT	≥ 4	R
**SNUDS-3**	Brain	*S. cohnii*	-	Cef-Pen-Oxa-Cip	≥ 4	R
**SNUDS-4**	Brain	*S. cohnii*	-	Cef-Pen-Oxa-Cip	≥ 4	R
**SNUDS-10**	Brain	*S. cohnii*	-	Oxa-Cip	0.5	R
**SNUDS-1**	Brain	*S. sciuri*	+	Oxa-Cip-Tet	≥ 4	R
**SNUDS-11**	Brain	*S. simulans*	-	Cip-Cli-SxT	≤ 0.25	S
**SNUDS-6**	Brain	*S. cohnii*	-	Cef-Pen-Oxa-Tet	≥ 4	R
**SNUDS-7**	Brain	*S. cohnii*	-	Tet	≤ 0.25	S
**SNUDS-8**	Trachea	*S. cohnii*	-	Cef-Pen-Oxa-Tet	2	R
**SNUDS-5**	Lung	*S. lentus*	-	Oxa-Cli	0.5	R
**SNUDS-9**	Brain	*S. sciuri*	-	Cef-Pen-Oxa-Tet	2	R
**SNUDS-13**	Brain	*S. cohnii*	-	Oxa-Cip	0.5	R
**SNUDS-14**	Lung	*S. cohnii*	-	Oxa-Cip-Tet-SxT	0.5	R
**SNUDS-12**	Brain	*S. lentus*	-	Oxa-Cip-Cli-QDA –SxT	0.5	R
**SNUDS-15**	Brain	*S. lentus*	-	Cef-Pen-Oxa-Cip-Tet-SxT	2	R
**SNUDS-16**	Trachea	*S. epidermidis*	-	-	≤ 0.25	S
**SNUDS-17**	Brain	*S. sciuri*	-	Oxa-Cip-Ery-Cli	0.5	R
**SNUDS-18**	Brain	*S. sciuri*	-	Oxa-Cip-Ery-Cli-Tet-SxT	2	R

^*^Cef: cefoxitin; *Pen*: penicillin; *Oxa*: oxacillin; *Cip*: ciprofloxacin; *SxT*: sulphamethoxazole/trimethoprim; *Tet*: tetracycline; *Cli*: clindamycin; *QDA*: quinupristin-dalfopristin; *Ery*: erythromycin.

### Antimicrobial susceptibility and detection of *mecA*

Among the 15 antimicrobials tested in this study, resistance against oxacillin (15 isolates, 83.3%), ciprofloxacin (12 isolates, 66.7%) and cefoxitin (7 isolates, 38.9%) were most frequently observed (Table 2). Among the 15 oxacillin resistant isolates, 11 isolates showed resistance against more than three antimicrobials other than oxacillin. Despite the high oxacillin resistance rate, only one isolate (SNUDS-1, *S. sciuri*) carried *mecA* (Table 2)*.* All isolates were susceptible against gentamicin, linezolid, mupirocin, quinopristin/dalfopristin, teicoplanin, trimethoprim/sulfamethoxazole and vancomycin.

### SCC*mec* typing

SCC*mec* typing was performed on SNUDS-1. The amplicon size of the *mec* complex was 1,963 bp and 804 bp when the mA7, mI6, IS7 and IS2 (iS-2) primers were used. According to Kondo *et al*. [18], this means SNUDS-1 carried *mecA*, *mecI* and *IS431* upstream of *mecA* but no *IS1272.* These features showed that SNUDS-1 possessed the class A *mec* complex.

The *ccrA* gene was successfully amplified from the genomic DNA of SNUDS-1 using primers for ccrA-UF1 and -UR1, ccrA-UF2 and -UR2 and ccrA_up and _dw pairs. The *ccrB* gene was also amplified with primers for ccrB-UF1 and -UR1 and ccrB_up and _dw pairs. However, the amplified products obtained using the primers for ccrC-UF1 and -UR1 and 28–24 and -26 pairs were determined as non-specific products by sequencing analysis. The amplified products obtained using ccrA-UF1 and -UR1 (1095 bp of final product) and ccrB-UF1 and -UR1 (1047 bp of final product) primers were sequenced and blast searched against other *ccr* genes using GenBank (http://blast.ncbi.nlm.nih.gov). The *ccrA* gene product was similar to the *ccrA3* from the *S. cohnii* strain WC28 (Acc. No. GU370073.2) with a similarity of 94%. The sequence of the amplified *ccrB* showed the closest match to the sequences of the *ccrB3* of *S. sciuri* strain MCS 24 (Acc. No. AB587080.1) with a similarity of 95%. Based on the combined results presented above, the isolate SNUDS-1 was shown to possess SCC*mec* type III; the type 3 *ccr* complex and the class A *mec* complex [19].

## Discussion

CoNS have been frequently detected from food animals [12,22], but hardly reported in ducklings. In this study, staphylococci isolated from the organs of duckings with tremors, such as the brain, were investigated. Central nervous system signs in avian species are usually caused by virus and mycotoxin [23]. This can be explained by the fact that there is an unusual cause of tremors from septicaemia and systemic infection caused by bacteria and candida [24,25]. Also, the staphylococci isolated in this study might be result from a secondary infection of predisposed viral diseases. Other bacterial species except Staphylococci were not isolated from organ samples.

Antimicrobial resistant of food animals is a serious public health problem because of the possibility of dissemination of the antimicrobial resistant bacteria to humans via food [26]. In Korea, extensive studies were performed to investigate the methicillin resistance in major food animals such as bovine raw milk, beef, pork and chicken meat [27,28]. For ducks, however, there are few studies although the South Korean duck industry has been growing fast recently [29]. Moreover, the possibility of spread from ducks to humans may be higher than that from chickens due to unapparent infections and poor sanitary conditions [30].

In this study, *mecA* was detected from only one out of 15 isolates showing oxacillin resistant phenotype. Although 10 of the *mecA*-negative oxacillin resistant isolates had MIC ≤2 μg/ml, 4 isolates had MICs over 4 μg/ml as the *mecA*-positive isolate. There are unusual MR-CoNS that have a resistance mechanism other than the production of PBP2a, which have been reported as borderline methicillin-resistant strains [31]. Most of them are resistant to oxacillin due to their plasmid-borne determinants, including hyperproduced penicillinases, genes conferring resistance to cadmium, or other gene products [32,33]. It is also possible that these *mecA-*negative oxacillin resistant CoNS possessed *mecA* alleles, which could not be detected by the primers used in this study. According to Monecke *et al*., many CoNS strains of animal origin show diversity in *mecA* sequences and have a different impact on β-lactam resistance [34].

In this study, the *mecA* carrying isolate, SNUDS-1 was shown to be of the SCC*mec* type III. According to Zong *et al*. [18], the common types of SCC*mec* in MR-CoNS are II, III, IV and V and Type III is common and widely distributed in a variety staphylococcal species. There are plenty of *ccr* variants with nucleotide differences and the allotype is assigned based on 85% identity. Since many new types of *ccr* allotypes have been reported, especially in MR-CoNS, multiple pairs of primers for the *ccr* genes should be used to maximize the possibility of detection [35].

Although the SCC*mec* type III is not the predominant type in human, MR-CoNS in food animals should be monitored to prevent the dissemination of antimicrobial resistance genes and resistant pathogens to the community.

## Conclusions

From this study, CoNS isolates from ducklings and their antimicrobial susceptibility were investigated. Resistance against oxacillin, ciprofloxacin and cefoxitin were most frequently observed and one strain carried the *mecA* gene which corresponds to a class A *mec* complex by SCC*mec* typing. Because of the possibility of dissemination of the antimicrobial resistant bacteria to human via the food processing chains, screening the antimicrobial resistance bacteria in duck industry should be performed further.

## Abbreviations

Ccr: Cassette chromosome recombinase; CoNS: Coagulase-negative staphylococci; J: Junkyard; PBP2a: Penicillin-biinding protein; SCCmec: Staphyloccoccal cassette chromosome *mec*; TSB: Tryptic soy broth.

## Competing interests

The authors declare that they have no competing interests.

## Authors’ contributions

JEH has been involved in drafting the article and carried out collection of the samples and interpretation of the data. SYH conceived the study and participated in its design and coordination, and helped to draft the article. JJK participated in the discussion on the study design. SPS and JWJ involved collection of the samples. JYC, YHP and SCP helped to draft the manuscript. All authors read and approved the final manuscript. All authors read and approved the final article.
